# Can we consider soluble herpes virus entry mediator (sHVEM) as a tumor marker?

**DOI:** 10.22088/cjim.13.4.693

**Published:** 2022

**Authors:** Seyed Mohammad Javadzadeh, Mohsen Tehrani, Mohsen Keykhosravi, Rajeeh Mohammadian- Amiri, Omolbanin Amjadi, Nasim Hafezi, Ehsan Zaboli, Mobina Montazeriun, Abolghasem Ajami

**Affiliations:** 1 Department of Immunology, Faculty of Medicine, Mazandaran University of Medical Sciences, Sari, Iran; 2 Molecular and Cell Biology Research Center, Mazandaran University of Medical Sciences, Sari, Iran; 3 Department of Infectious Diseases, Antimicrobial Resistance Research Center, Mazandaran University of Medical Sciences, Sari, Iran; 4 Gastrointestinal Cancer Research Center, Non-Communicable Diseases Institute, Mazandaran University of Medical Sciences, Sari, Iran; 5 Gastrointestinal Cancer Research Center, Mazandaran University of Medical Sciences, Sari, Iran

**Keywords:** Herpesvirus entry mediator, Gastric cancer, Breast cancer, colorectal cancer

## Abstract

**Background:**

Immune checkpoint molecules have critical roles in directing immune responses into co-inhibitory and co-stimulatory signals. Herpes virus entry mediator (HVEM) is a receptor of tumor necrosis factor receptor superfamily with unique features due to its interaction with both inhibitory and stimulatory ligands. The aim of this study was to measure the serum level of the soluble form of HVEM in patients with gastric, colorectal and breast cancers and evaluating its diagnostic and prognostic value.

**Methods:**

The concentration of the soluble HVEM (sHVEM) was determined in the serum of 36 patients with breast cancer, 50 patients with colorectal cancer and 59 patients with gastric cancer using ELISA method. Moreover, 50 healthy donors (HD) as well as 31 patients with non-ulcer dyspepsia (NUD) were used as control groups. The patients’ samples were obtained from the Biobank of Cancer Research Center, Mazandaran University of Medical Sciences, Sari, Iran.

**Results:**

The level of sHVEM was significantly higher in patients with gastric (P=0.001) and breast cancer (P=0.01) than in control groups (HD). The higher level of sHVEM was observed in colorectal cancer patients in comparison with HD group, although it was not significant. Moreover, the elevated level of sHVEM was shown to be higher significantly in stage III and IV compared to stage I and II in breast cancer (P=0.03). Similar finding was detected in gastric and colorectal cancers, but not to be statistically significant.

**Conclusion:**

The results of the present study suggest that the serum level of sHVEM may be considered as a promising indicator for diagnosis as well as evaluating the progression of cancers such as gastric, breast and colorectal cancers.

Cancer is known as the second cause of death worldwide, preceded by cardiovascular diseases (1). In the U.S alone, it is estimated that 1.8 million individuals will be diagnosed with cancer among whom 606520 will die during 2020 (2). Hence, more efforts still need to be made to find out new strategies for cancer diagnosis and treatment. The immune system as the guardian against non-self-agents plays a pivotal role in eliminating tumor cells. Hence, cancer cells exploit various mechanisms enabling them to escape the immune response which is essential for tumor progression. (3, 4). Immune checkpoints are regulatory molecules that modulate the immune systems and can be involved in tumor immune escape. The study of the molecules within the immune checkpoint framework is essential for identifying novel treatment targets, improving the cancer immunotherapy, and comprehending the diagnostic and prognostic value of these molecules (5). Upon interaction with their receptor/ligands, they trigger cascades of signaling pathways leading to stimulation or inhibition of the immune response (6).

Recently, HVEM (Herpesvirus entry mediator), which mediates the entry of the herpes simplex virus into the cells, has reported as an immune checkpoint molecule. HVEM, also called tumor necrosis factor receptor superfamily 14 (TNFRSF14), was first identified as a ligand for the herpes virus glycoprotein D (5, 7). It is expressed on non-lymphoid organs such as lung, liver, and kidney as well as on several types of hematopoietic cells including T-, B-, NK-, dendritic-, and myeloid- cells (8). HVEM can induce different immune responses depending on the type of receptor/ligand it interacts with. Binding of HVEM with BTLA (B- and T-lymphocyte attenuator) and CD160 triggers the inhibitory signals leading to suppression of T cells while its ligation with LIGHT (the lymphotoxin-like inducible protein that competes with glycoprotein D for herpes virus entry on T cells) and LTα (lymphotoxin-α) results in activation of T lymphocytes (6).

Several studies have shown that HVEM may help the progression of tumors via influencing both cancer cells and immune cells. The silencing of the HVEM inhibited the proliferation of tumors in cancers of colorectal and esophagus. On the other hand, expression of HVEM was shown to be correlated with immune downregulation as it was observed through reduced expression of interferon ɣ, perforin, and granzyme B (5, 8-10). 

The role of HVEM in tumor progression is complicated and, in some cases, contradicting. For example, HVEM/BTLA pathway was shown to inhibit the production of cytokines and the proliferation of tumor antigen-specific CD8^+^ T cells (11). Besides, the BTLA/HVEM pathway was reported to result in the suppression of immune responses by recruiting the tyrosine phosphatase and transmitting the inhibitory signals (12). This inhibitory function was further highlighted by studies which showed that suppression of this pathway that led to the enhancement of the immune response and inhibition of tumor proliferation (10, 13-15). On the contrary, the interaction between HVEM and LIGHT is suggested to trigger a positive costimulatory signal, leading to the initiation of the inflammatory responses (12). For instance, ligation of LIGHT to HVEM has been shown to stimulate the production of chemokines and induces apoptosis in B-CLL (chronic lymphocytic leukemia) cells (16). In addition to the membrane HVEM (mHVEM), the level of the serum soluble HVEM (sHVEM) could be correlated with disease severity and progression. Now a little information is available on the changes of serum level and function of sHVEM in malignancies. High serum level of sHVEM has been observed in some cancers including hepatocellular carcinoma (HCC) and cutaneous T-cell lymphoma with clinicopathological importance (17, 18). The aim of the present study was to evaluate the serum level of sHVEM in patients with breast cancer (BC), colorectal cancer (CRC), and gastric cancer (GC) to find out the possible relationship between the concentration of sHVEM with the presence and severity of cancers.

## Methods

In the present study, a total of 145 patients with cancer were enrolled. The patients were confirmed based on established clinical, radiological, endoscopic, and histological criteria as gastric, breast, and colorectal cancers in the Department of Oncology of Imam Hospital (Mazandaran University of Medical Sciences, Sari, Iran). The number of patients in each group accounted for 36 patients with BC, 50 patients with CRC, and 59 patients with GC. Thirty-one patients with non-ulcer dyspepsia (NUD) were considered as the control group for GC. Moreover, 50 matched normal serums were collected from healthy donors (HD) serving as the control group. The sera of patients were obtained from the Biobank of Cancer Research Center, Mazandaran University of Medical Sciences, Sari, Iran. The stage of tumors was determined according to the TNM /UICC guidelines. The serum of healthy controls was collected from the venous blood of healthy individuals that referred to the Iranian National blood transfusion center, Sari, Iran.

**Enzyme Linked Immunosorbent Assay (ELISA): **The concentration of the secreted HVEM was assessed by ELISA method (R&D, Minneapolis, MN). Mouse anti-human HVEM capture antibody was diluted to 2 µg/ml and added (100 µl) to each well of 96-well microplates and incubated at 4 ˚c overnight. After washing (0.05% tween 20-PBS), the wells were blocked with 1% BSA in PBS and incubated for 2 hours at room temperature. After washing, the patients and control sera (1/20 diluted by PBS-%0.05 tween 20) were added to the wells and incubated at room temperature for 2 hours. Then, biotinylated Goat anti-human HVEM detection antibody was added to the wells for 1 hour at room temperature. Following the washing step, 100 µl of Streptavidin-HRP with concentration of 100 ng/ml was added and incubated for 20 minutes at room temperature. After washing, substrate solution (TMB) was added and the reaction was then stopped by adding the stop solution (H2O2). The optical density (OD) of each well was determined, using a microplate reader at wavelength of 450nm and 540nm (as reference wavelength). The specificity and sensitivity of this assay was determined at 50 ng/ml and 15 pg/ml, respectively.

**Statistical Analysis: **Data were analyzed using GraphPad Prism Version 6 for windows (GraphPad Software, San Diego, CA, USA). Parametric or nonparametric distribution was determined by Kolmogorov–Smirnov test. Difference in HVEM concentration between the study groups were tested using Mann–Whitney U test. Data were expressed as means and standard deviation (SD). A p<0.05 was considered to be statistically significant.

## Results

**Figure 1:**
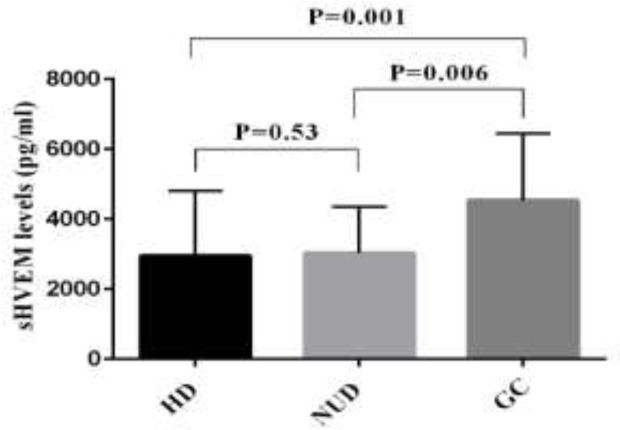
Serum soluble herpes virus entry mediator levels (sHVEM) level in gastric cancer (GC) versus non-ulcer dyspepsia (NUD) and healthy donors (HD)

**Figure 2:**
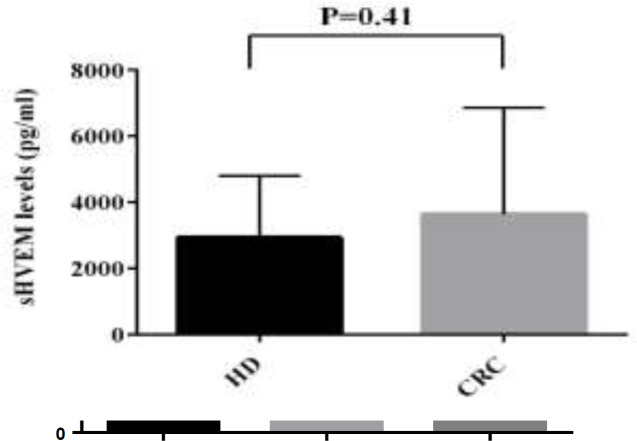
Serum sHVEM levels in colorectal cancer (CRC) and breast cancer (BC) versus HD

**Figure 3:**
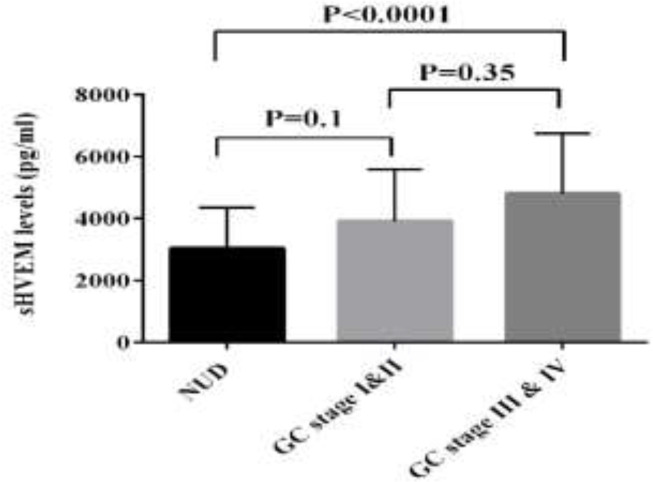
Serum sHVEM levels in GC stages versus NUD

**Figure 4:**
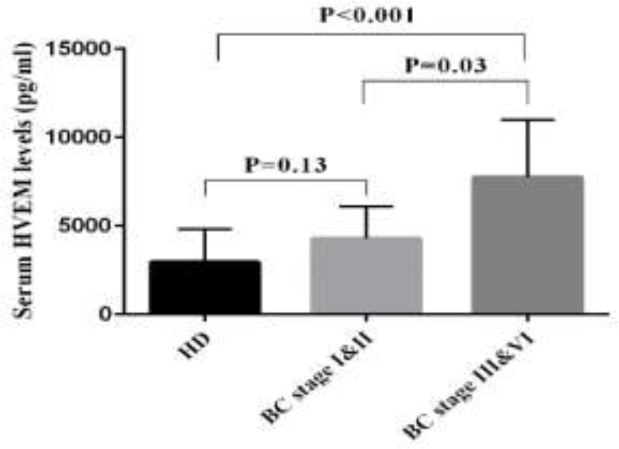
Serum sHVEM levels in CRC stages versus HD

**Figure 5:**
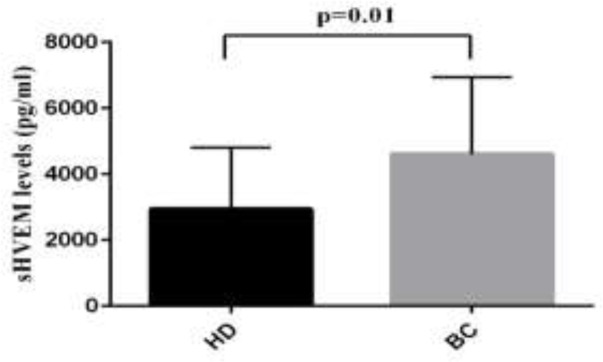
Serum sHVEM levels in BC stages versus HD

The level of sHEVM was evaluated in the sera of 50 patients with CRC, 36 patients with BC and 59 patients with GC by ELISA. The mean levels of sHVEM were significantly higher in GC patients compared to NUD group (mean±SD; 4528±1915 versus 3028±1319; P=0.006). Similarly, comparing to the HD group, the GC patients showed elevated levels of sHVEM (mean±SD; 4528±1915 versus 2946±1857*; *P=0.001). However, the concentration of sHVEM was not significantly different between NUD and HD groups (table 1 and figure 1). The comparison between BC patients and control group revealed a significant increase in the concentration of sHVEM in BC patients (mean±SD; 4612±2329 versus 2946±1857; P=0.01). Although the concentration of sHVEM raised in CRC patients compared to the control group, this change was not statistically significant (table 1 and figure 2). The association between demographic features of these patients and the level of HVEM is described in table 2. In patients with BC, the serum level of sHVEM was significantly higher in stage III and IV compared to stages I and II. Although not significant, the level of sHVEM was higher in patients with stages III and IV of CRC and GC compared to those with stage I and II (figures 3, 4 and 5). Furthermore, comparing to the younger individuals, the BC patients older than 60 years of age showed the higher levels of sHVEM. Moreover, in CRC patients, the level of sHVEM increased in those older than 60 years. However, this difference was not significant. In GC patients, no age-related changes of sHVEM level were observed. Additionally, we did not observe any significant difference between family history and serum sHVEM in colorectal cancer patients. 

**Table 1:** Comparison the serum level of HVEM in gastric, colorectal and breast cancer groups

Group	Number HVEM (mean± SD) pg/ml	P -value
**NUD** **Gastric cancer**	31 3028±1319 59 4528± 1915	0.006
**HD** **Gastric cancer**	50 2946±1857 59 4528±1915	0.001
**HD** **NUD**	50 2946±1857 31 3028±1329	0.53
**HD** **Colorectal cancer**	50 2946±1857 50 3651±3226	0.41
**HD** **Breast cancer**	50 2946±1857 36 4612±2329	0.01

NUD: Non-ulcer dyspepsia, GC: Gastric cancer, CRC: Colorectal cancer, BC: Breast cancer, HD: Healthy donor

**Table 2:** Association of HVEM expression with clinicopathological factors

Patient group	Variables	Number HVEM (mean± SD) pg/ml P-value
** CRC**	Age ≥60 <60	n=21 6418±5753 0.22 n=29 2777±1149
	TNM stage I-II III-IV	n=24 3000±1638 n=18 5109±5434 0.56
	Family History Yes No	n=28 3101±1456 n=20 3011± 1290 0.9
** GC**	Age ≥60 <60	n=49 4495±1828 0.9 n=10 4692±2401
	TNM stage I-II III-IV	n=20 3911±1673 0.35 n=33 4806±1935
	Family History NA*	
** BC**	Age ≥60 <60	n=9 7046± 3081 0.02 n=27 4761±2415
	TNM stage I-II III-IV	n=22 4281±1802 0.03 n=12 7758±3232
	Family History NA*	

## Discussion

In this study, we showed the level and clinicopathological significance of serum sHVEM in patients with gastric, colorectal and breast cancers. Results of the study demonstrated a significant increase in the level of sHVEM in GC patients compared to the NUD and HD controls. Furthermore, the elevated levels of sHVEM were shown to be directly associated with the advanced stages of the diseases. Our findings are in line with those of Sook-Kyoung Heo et al. who showed that patients with GC have increased levels of serum HVEM and decreased expression of mHVEM on leukocytes. They showed that activation of leukocytes in the presence of proinflammatory stimulants leads to reduced level of membrane HVEM and increased level of sHVEM (12). On the other hand, increased level of membrane HVEM and BTLA was reported in GC tissues and was shown to be associated with worse outcome including enhancement of invasion, metastasis and low survival (19). This suggests that in addition to the tumor-derived HVEM, the serum level of HVEM is associated with poor prognosis in GC patients. In our study, comparing to the HD group, BC patients showed elevated levels of sHVEM which was more evident in late stages of the disease. Hence, the level of sHVEM can be considered as a potential marker for diagnosis of patients with the end stages of breast cancer.

Until we know, only the study by Tsang et al. evaluated HVEM expression status in BC. They identified overexpression of mHVEM in breast cancer cells, mainly HER2-OE subtypes. On the other hand, the high levels of HVEM was demonstrated to be associated with more aggressive stages of the disease, low tumor infiltrating lymphocytes and low survival. (5). In addition, an association found between HVEM gene polymorphism and the risk of sporadic breast cancers, suggesting its relevance in the process of breast carcinogenesis (20).

 Although not significant, CRC patients showed an increased level of sHVEM, especially in late stages when they were compared to the HD group. Several studies have examined the expression of mHVEM in CRC biopsies. Inoue et al. observed a high expression of mHVEM in CRC tissues compared to normal colonic epithelial cells with higher expression in advanced stages.

Additionally, HVEM status independently predicted CRC outcomes. (9). Moreover, overexpression of mHVEM has been observed in liver biopsies of patients with colorectal liver metastasis. Patients with higher HVEM expression exhibited worse overall survival and low tumor-infiltrating lymphocytes (21). 

Same as our study, the level of sHVEM has also been evaluated in some other cancers. For example, an increased level of sHVEM and sLIGHT was reported in progressive cutaneous T-cell lymphoma. Furthermore, a positive correlation has been found between sHVEM and sLIGHT level, as well as between sHVEM and Th-2 inducing cytokines such as IL-4 and IL-10 (17). Similarly, an increased level of serum sHVEM was observed in hepatocellular carcinoma, which was associated with progressive stages of the disease (18). Besides, it is suggested that serum sHVEM may serve as an indicator for evaluating the disease severity as well as response to treatment in lung cancer (22). 

Most of the studies focused on the evaluation of HVEM expression in tumor tissues and found a correlation between HVEM overexpression and tumor progression (23, 24). However, the present study demonstrates that the level of sHVEM can also increase in the serum of patients with GC, CRC and BC, which is more remarkable in advanced stages. This indicates that the serum level of HVEM is associated with the occurrence and progression of cancers and can serve as a potential tumor marker for cancer diagnosis, especially those with poor prognosis. Nevertheless, further evaluations, including the effect of sHVEM level on the recurrence, metastasis, and survival of patients should be performed to confirm the diagnostic and prognostic value of serum HVEM in cancers. On the other hand, understanding the role and mechanism of sHVEM in the suppression of anti-tumor immune responses might be effective in reducing tumor progression. 
